# On-Demand Bioadhesive Dendrimers with Reduced Cytotoxicity

**DOI:** 10.3390/molecules23040796

**Published:** 2018-03-30

**Authors:** Feng Gao, Ivan Djordjevic, Oleksandr Pokholenko, Haobo Zhang, Junying Zhang, Terry W. J. Steele

**Affiliations:** 1 School of Material Science and Engineering, Beijing University of Chemistry Technology, North Third Ring Road 15, Chaoyang District, Beijing 100029, China; gaofeng@mail.buct.edu.cn (F.G.); zhanghaobo9093@163.com (H.Z.); 2 Escuela de Ingeniería y Ciencias, Tecnologico de Monterrey, Ave. Eugenio Garza Sada 2501, Monterrey 64849, NL, Mexico; idjordjevic@ntu.edu.sg; 3 School of Materials Science and Engineering, Division of Materials Technology, Nanyang Technological University, Singapore 639798, Singapore; opokholenko@ntu.edu.sg

**Keywords:** PAMAM, dendrimers, bioadhesive, diazirine

## Abstract

Tissue adhesives based on polyamidoamine (PAMAM) dendrimer, grafted with UV-sensitive aryldiazirine (PAMAM-g-diazirine) are promising new candidates for light active adhesion on soft tissues. Diazirine carbene precursors form interfacial and intermolecular covalent crosslinks with tissues after UV light activation that requires no premixing or inclusion of free radical initiators. However, primary amines on the PAMAM dendrimer surface present a potential risk due to their cytotoxic and immunological effects. PAMAM-g-diazirine formulations with cationic pendant amines converted into neutral amide groups were evaluated. In vitro toxicity is reduced by an order of magnitude upon amine capping while retaining bioadhesive properties. The in vivo immunological response to PAMAM-g-diazirine formulations was found to be optimal in comparison to standard poly(lactic-*co*-glycolic acid) (PLGA) thin films.

## 1. Introduction

**Figure 1:**
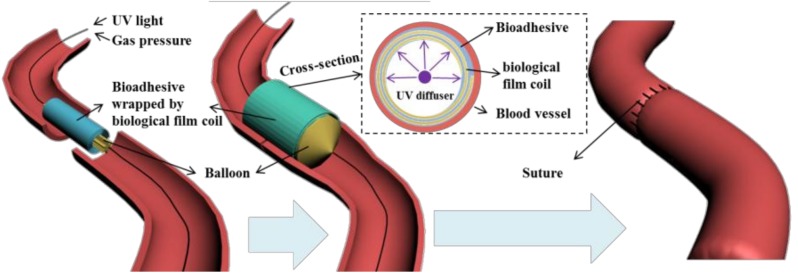
Proposed strategy for blood vessel fixation: UV crosslinked adhesive hydrogel is spread on the surface of bio-degradable film coil, wrapped around the balloon stent. Adhesives layer is in direct contact with blood vessel once the coil is expanded by balloon inflation. UV light is transferred through the stent and crosslinks the adhesive layer with the help of a light diffuser.

Adhesive hydrogels generated by crosslinking of polymers are designed to disperse mechanical stress and allow fluid transport through tissue interfaces [1]. Such formulations are considered to be useful to replace the suturing of delicate tissues and to avoid tissue piercing. This feature is particularly important for vascular anastomosis (Figure 1) where adhesive formulations should allow strong tissue fixation in order to replace traditional suturing fixation methods [2,3,4,5].

**Figure 2:**
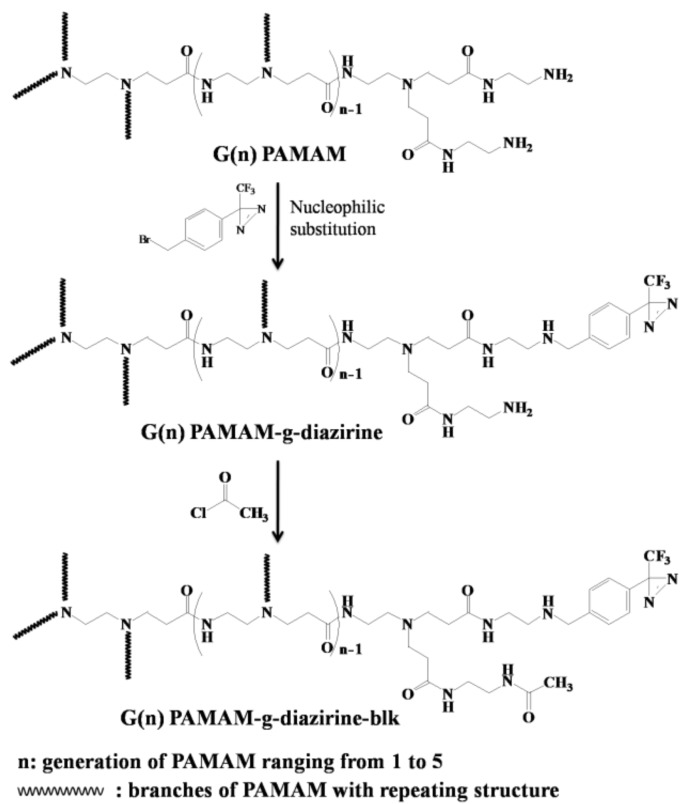
Synthesis pathway for PAMAM-g-diazirine and PAMAM-g-diazirine-block: the first step is conjugation of aromatic diazirine onto G1–G5 PAMAM macromolecules; the second step is the reaction between free –NH_2_ groups and acetyl chloride resulting in a reduced number of free –NH_2_ groups that can cause cytotoxicity.

Although developed for more than half a century, non-invasive tissue fixation strategies still rely on cyanoacrylate [6] and the fibrin glue formulations [7]. Cyanoacrylate, also known as ‘super glue’, is able to generate reliable adhesion strength towards the surface of tissue after an in-situ crosslinking process in the presence of moisture [8]. However, the heat generated during the polymerization can burn the tissue [9] and the toxic degradation products limit the applications of cyanoacrylates to topical tissue fixations [6]. Fibrin glue is a protein-based adhesive that sets the adhesion strength through physical crosslinks with tissue interfaces of ~0.5 N/cm^2^. The in-situ crosslinking processes and swelling behavior of fibrin glue were found to be challenging for the hydrated tissue environment [7]. Tissue adhesives need to be able to bond both natural materials and synthetic films for applications involving implantable biosensors and drug depots [10,11,12,13,14,15,16,17,18,19,20]. Formation of covalent bonds between thin film or hydrogel adhesive formulations and soft tissue substrates is a promising strategy to achieve a reliable adhesion strength [3,15,16,19]. This can be achieved by diazirine-functionalized polymer formulations that crosslink under low energy UV light or voltage as previously published by our group [21,22,23,24]. Diazirines can be covalently attached on a PAMAM backbone through the reaction with pendant amine (–NH_2_) groups in a highly controlled manner. PAMAM-g-diazirine formulations resulted in tunable shear moduli of 1–100 kPa and maximum shear adhesion strength of 27 kPa when attached on wet, ex-vivo arteries, which is a promising in comparison to fibrin adhesives [25]. In vitro 3T3 fibroblasts cell culture test showed no leaching of potentially toxic components from the crosslinked adhesive. It should be noted that significant cytotoxicity was caused by the uncrosslinked parts of macromolecules and their inherent –NH_2_ groups from the PAMAM dendrimer [21,22,24]. Positively charged amines are known to disrupt cell membranes [16]. In order to avoid direct contact of PAMAM with cells we have mediated the cytotoxic influence of –NH_2_ groups by chemical capping. This is achieved by reacting free –NH_2_ groups of PAMAM with acetyl chloride, which produces a neutral dendrimer free of cationic charge (Figure 2).

The number of functional groups doubles during the divergent synthesis of PAMAM with each generation and the diameter of the molecule extends by only 1 nm [26]. Surface functional groups of PAMAM dendrimers exponentially increases with the next generation, likely correlating with toxicity. The work presented herein is based on the following hypotheses: (i) tissue adhesion is caused by UV-activated crosslinking of diazirine end-groups and the adhesion strength is dependent on diazirine concentration and not dendrimer size; (ii) the presence of protective amide groups (Figure 2) will not interfere with carbene crosslinking; and (iii) capping of –NH_2_ groups (PAMAM-g-diazirine-blk) decreases both in vitro toxicity, and in vivo immunological response. The conjugation of acetyl chloride onto pendant –NH_2_ groups of PAMAM reduces or removes the cationic charge, mediating cationic cytotoxicity without significantly lowering intermolecular crosslinking. PAMAM dendrimers (G1 to G5) were conjugated with diazirine (PAMAM-g-diazirine) and acetyl chloride (PAMAM-g-diazirine-blk) in order to examine new formulations both in vitro and in vivo [22,27]. Prior to biocompatibility tests, the conjugated dendrimer structures were characterized with size exclusion chromatography (SEC), nuclear magnetic resonance (^1^H-NMR) spectroscopy and amino-group quantitation. Dynamic mechanical properties of dendrimer adhesive formulations were monitored by photorheometry in real time in order to determine the dose dependent moduli.

## 2. Results and Discussion

**Figure 3:**
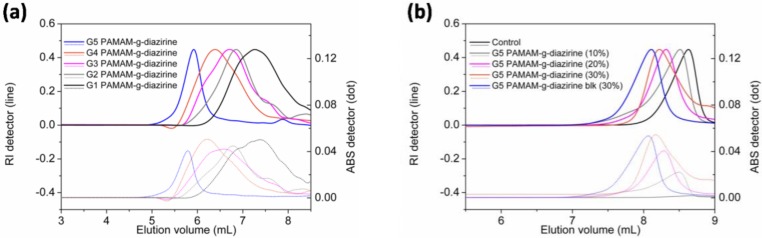
SEC-MALS-UV analysis of conjugated PAMAM dendrimers: (**a**) chromatographs of G1–G5 PAMAM-g-diazirine (solid lines stand for the normalized refractive index, and the corresponding dashed lines were the UV absorption caused by the conjugated diazirine on each conjugates injected); (**b**) chromatographs of G5 PAMAM-g-diazirine and its ‘blocked’ conjugate with acetyl chloride.

Diazirine was conjugated on G1–G5 PAMAM via nucleophilic substitution reactions as demonstrated in Figure 2 and the formulations were analyzed for their molecular weight (SEC; Figure 3). Neat PAMAM macromolecules (G1–G5) were used as controls (Figure 3a). The solid lines in Figure 3a present the refractive index signals and are shifted from right to left indicating the increase of molecular weight with increasing dendrimer generation (G1 to G5). Dashed lines in Figure 3b represent the corresponding UV absorption at 350 nm caused by the conjugated diazirine. The UV absorption correlated with the increase of diazirine conjugation percentage (Table S1, see Supplementary Section). Similar to our previous work, diazirine conjugation onto PAMAM macromoloecules is obtained with a high degree of control over the percentage of conjugation (Figure S1). G1–G5 PAMAM-g-diazirine conjugates with capped amines (Figure 2) were also analysed by SEC and compared to PAMAM-g-diazirine as presented in Figure 3b. As expected, the acetyl chloride blocking caused the increase of molecular weight of the conjugates as the peak value of refractive index signal of G5 PAMAM-g-diazirine-blk (blue) occurred earlier than that of G5 PAMAM-g-diazirine (Figure 3b).

**Figure 4:**
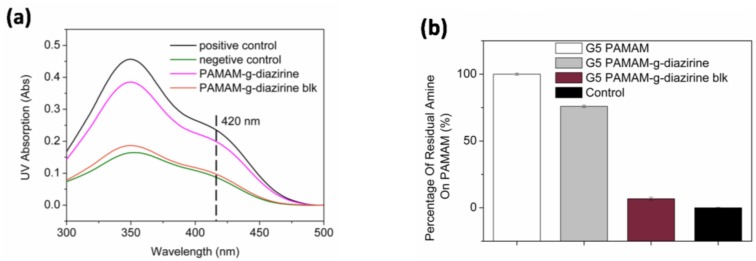
Primary amine concentration from PAMAM adhesive formulations analyzed with TNBS assay: (**a**) UV absorption curves of TNBS for the G5 PAMAM and its conjugates (pure G5 PAMAM was tested as positive control and negative control was 0.1 M NaHCO3 buffer; (**b**) Percentage of residual –NH_2_.

The percentage of residual non-reactive –NH_2_ groups was calculated based on the increase of the molecular weight evaluated from the elution volume (Table S1; Supplementary Section). In order to confirm the concentration of free amines, all formulations were quantified for free amines with the TNBS assay. TNBS reacts with –NH_2_ groups in aqueous solution forming a complex with a maximum UV absorbance at 420 nm. The residual amine was examined by comparing the UV absorption at 420 nm of each conjugate (Figure 4). Note that only 6% of –NH_2_ groups were detected for G5 PAMAM-g-diazirine-blk. Diazirine conjugation percentages were also calculated from TNBS results. For example, the residual free –NH_2_ percentage was 75% for G5 PAMAM-g-diazirine, indicating that 25% of the –NH_2_ groups were replaced by aromatic diazirines during the conjugation reaction (Table S1).

**Figure 5:**
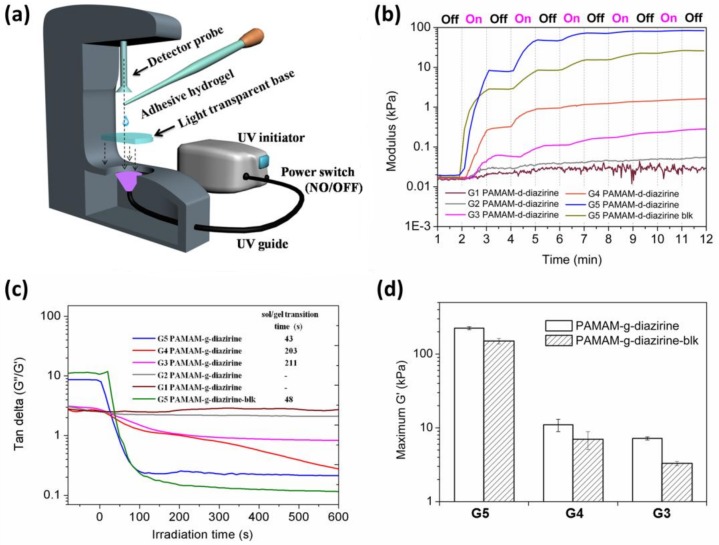
Photo-rheometry of PAMAM-g-diazirine adhesives: (**a**) schematic illustration of rheometry measurements with UV activation; (**b**) dynamic change of storage modulus (G’) and loss modulus (G”) with UV activation (diazirine conjugation percentages were 20% for G2–G5 and 37.5% for G1; concentration for all conjugates was 50 wt % in PBS); (**c**) ratio between G’ and G” under continuous UV activation (inset: sol/gel transition time); (**d**) maximum G’ obtained for each conjugate.

The mechanical properties of PAMAM-g-diaziridene conjugates were monitored in real time with photorheometry as presented in Figure 5a. Dendrimer adhesive solution was placed in between the light transparent base and the rheometer probe and UV light was introduced by a waveguide (Figure 5a). 

The irradiation intensity on the sample was adjusted to a constant value of 20 ± 2 mW cm^−2^ in all experiments. The curves in Figure 5b demonstrate the relationship between the storage moduli and irradiation (UV activation) time for G1–G5 PAMAM-g-diazirine. Stepwise shaped curves were detected for all the conjugates as well as the G5 PAMAM-g-diazirine-blk after several 1 min “ON/OFF” intervals of UV activation. The crosslinking process started immediately when UV light was ‘ON’ leading to a rapid increase of the storage modulus (G’) and the loss modulus (G”). The crosslinking reaction stops immediately when UV was switched “OFF” thus resulting in flat line of G’ vs. time (Figure 5b). This behavior is a result of instant, non-specific covalent insertion of carbenes without chain propagation during the crosslinking process [28]. Tunable and highly controllable mechanical properties are evident from our dendrimer adhesives, thus reducing the risks of substrate-adhesive modulus mismatch, potentially reducing the chances of adhesive failure under dynamic strains [29,30]. 

The relationships between G”/G’ (tan delta) under continuous UV stimulation time for G1–G5 PAMAM-g-diazirine and G5 PAMAM-g-diazirine-blk are plotted in Figure 5c. All these ratios were greater than 1 before UV exposure, indicating the adhesives were viscous liquids. The liquid consistency enabled adhesives to wet and fill irregular surfaces of soft tissue before the sol/gel transition (when G”/G’ > 1). G”/G’ values for G1 and G2 PAMAM-g-diazirine (G2, 30% diazirine conjugation percentage, G1, 62.5% diazirine conjugation percentage) never fell below 1, indicating incomplete crosslinking. Lower order PAMAM generations reduce the surface functional group concentration and dendrimer to dendrimer interpenetration entanglement as well. Thus, the intermolecular crosslinking caused by carbene was insufficient for the formation of an elastic 3D network when the generation was lower than G3. The cohesive failures of G4 and G5 PAMAM-g-diazirine showed porous structures resulted from the formation of nitrogen indicating the consumption of diazirine.

The time taken by sol/gel transition increased from 43 s to 211 s when the generation of PAMAM-g-diazirine dropped from G5 to G3 (Figure 5c inset). Note that there was a five-fold drop of the sol/gel transition time from 43 s to 203 s when the dendrimer generation increased from G4 to G5. The modulus of G4 PAMAM-g-diazirine reached maximum of 10 kPa, about 20× lower than G5. In terms of biomimetic mechanical integrity G1–G4 PAMAM-g-diazirine are recommended for subcutaneous adipose tissue G’ ≈ 10 kPa [31] or other soft tissues with matching moduli. In terms of control over adhesion strength, G5 PAMAM-g-diazirine could be crosslinked on demand to meet requirements of treated tissues by simple reduction of UV energy used for crosslinking.

According to previous research, the branches of PAMAM above G5 were densely packed, thus forming spherical shapes in solution [32]. The morphology of PAMAM from G1 to G4 is planer and elliptical [33] and therefore the branch interpenetration intensity for G5 is higher than those of G4 and G3. As a consequence, we detected a different mechanical profile for G4 and G5 PAMAM-g-diazirine, which may be explained by the close packed geometry. Figure 5d demonstrates how after 5 min of UV activation G’ reached 225 kPa for G5 which were almost 20 times higher than that of G4 PAMAM-g-diazirine. Diazo functional group, also produced upon diazirine activation) is highly reactive towards nucleophiles such as –NH_2_ groups on the surface of PAMAM-g-diazirine and this allows specific crosslinking versus non-specific carbene insertion where the solvent competes with intermolecular bonding. Removal of amines and thus diazo-specific crosslinking may explain the decreased storage modulus (G’) of blocked PAMAM-g-diazirine presented in Figure 5d. The moduli change upon acetyl chloride blocking of G5 PAMAM-g-diazirine resulted in 225 kPa versus 151 kPa for amine capped. This value is still comparable to moduli of the soft tissues such as blood vessels (Table S2, supplementary section).

**Figure 6:**
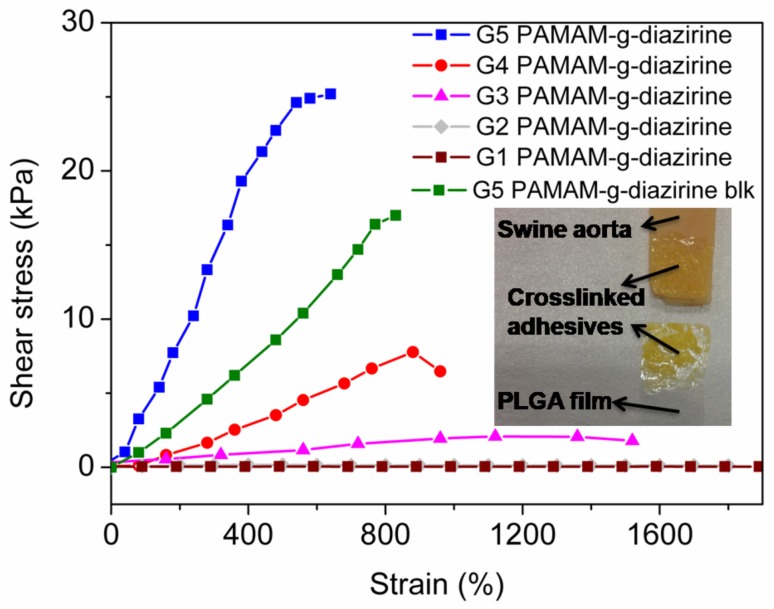
Ex-vivo lap shear adhesion test for PAMAM-g-diazirine bioadhesive formulations (inset: the ‘sandwich’ PLGA-adhesive-tissue structure and demonstration of cohesive failure.

As the primary evaluation method for adhesion performance of adhesives for soft tissues (Figure 1) [8] a lap shear test was performed ex-vivo with the endothelial surface of swine aorta procured from a local abattoir [34]. Conjugates with different formulations were prepared in PBS solution and sandwiched between PLGA film and fresh swine aorta. UV irradiation (5 min, 6 joules 365 nm) was applied for each group to ensure complete activation of conjugated diazirine. The stress/strain curves were recorded and plotted in Figure 6. The fracture occurred in the bulk of the adhesive indicating cohesive failure (Figure 6 inset) which is consistent with our previously reported results [21,22]. The adhesive strength decreased in the order of: G5 = 25.3 kPa, G4 = 7.4 kPa, and G3 = 3.4 kPa and this decrease in strength is consistent with the rheology data (Figure 5). The adhesive strength of G5 PAMAM-g-diazirine-blk was 17.1 kPa which is around 30% lower in comparison to the unblocked dendrimer formulation. It should be noted that the adhesion strength for G5 PAMMA-g-diazirine-blk is still significant higher than that of fibrin glue (5 kPa) [35]. Apart from relatively high adhesion strength, blocked dendrimer adhesive formulation is expected to significantly reduce cytotoxicity in vitro and in vivo.

**Figure 7:**
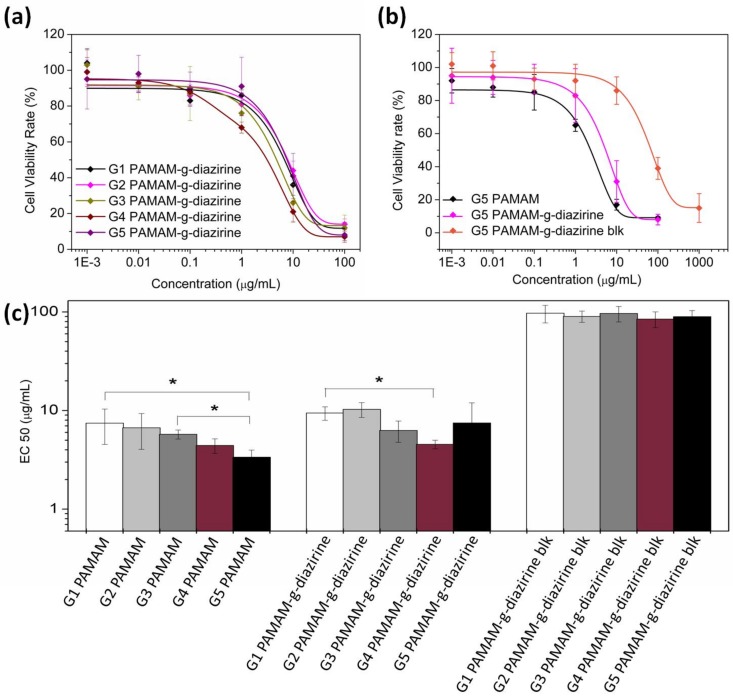
3T3 fibroblasts cytotoxicity of PAMAM-g-diazirine (G2–G5) 20% conjugates and 37.5% conjugate of PAMAM G1, with DMEM as negative control: (**a**) cell viability in contact with G1–G5 PAMAM-g-diazirine; (**b**) cell viability comparison between neat G5 and blocked G5 PAMAM-g-diazirine; (**c**) EC50 values for PAMAM, PAMAM-g-diazirine and PAMAM-g-diazirine-blk.

The potential cytotoxicity of adhesive formulations was evaluated as the half maximal effective concentration (EC_50_) of each formulation towards 3T3 fibroblasts cells. The cells were cultured with adhesive dendrimer solution and cell viability was evaluated with an Alamar Blue assay by comparing the cell reductivity with the negative control. Figure 7a,b show the cell viability when cultured with PAMAM-g-diazirine from G1 to G5 (the diazirine conjugation percentage was 30% for G2 to G5 and 37.5% for G1). EC50 values for each generation were fitted and plotted in Figure 7c. EC50 increased significantly from 2.4 μg/mL (G5) to 7.3 μg/mL (G1), indicating the decrease of cytotoxicity from reduced cell exposure to –NH_2_ groups. Fibroblast viability rate was enhanced significantly for blocked PAMAM-g-dizairine formulations thus confirming the necessity of –NH_2_ capping (Figure 2). The EC_50_ values for G1–G5 PAMAM-g-diazirine-blk are almost 15 times higher than the corresponding values of the PAMAM-g-diazirine. 

**Figure 8:**
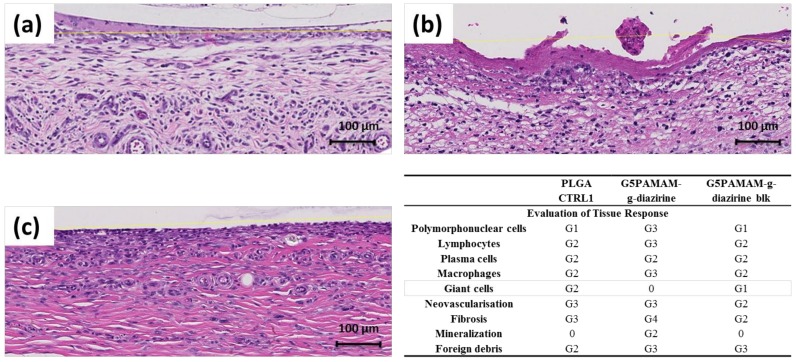
Histology results after 7 days of subcutaneous implantation in vivo by H&E staining methods: (**a**) PLGA control; (**b**) PAMAM-g-diazirine (15% diazirine conjugation, 75% wt % in PBS); (**c**) PAMAM-g-diazirine-blk (15% diazirine conjugation, 75% wt % in PBS); (**d**) immunological response evaluation: 0: No abnormalities detected (NAD); G1: minimal; G2: mild; G3: moderate; G4: marked; G5: severe.

PAMAM-g-diazirine conjugates were investigated in vivo in a subcutaneous implant animal model as displayed in Figure S2. Representative histology results are presented in Figure 8 and they show that all the implants (PLGA control, Figure 8a; and crosslinked adhesive formulations, Figure 8b,c) were surrounded by mononuclear cells (macrophages, lymphocytes and plasma cells), polymorphonuclear cells (mainly neutrophils), giant cells, fibrosis and neovascularization. The immunological response was more pronounced towards PAMAM-g-diazirine crosslinked formulations compared to PLGA control (Figure 8d).

Higher number of polymorph nuclear cells, lymphocytes, plasma cells and macrophages were detected along with intense fibrosis and neovascularization that was found on the interface with bioadhesive formulations (Figure 8b,c). As anticipated, mild immunological response was detected for blocked PAMAM-g-diazirine in comparison to neat formulation. This result is consistent with in vitro cytotoxicity of the same formulation (Figure 7) and is most likely a consequence of the cationic charge of pendant –NH_2_ on the interface between PAMAM-g-diazirine adhesive and the subcutaneous tissue.

Dendrimer toxicity is generally reported as hemolytic toxicity, cytotoxicity and hematological toxicity [36] Positively charged surface groups of PAMAM are known to destabilize negatively charged cell membranes causing lysis [37] Processes like adsorptive endocytosis and paracellular transport are the major cause for PAMAM dendrimers to cross cell membranes with the aid of primary amine groups [38]. As a result, the degree of cytotoxicity is determined by the concentration of –NH_2_ groups and therefore dendrimer-induced cytotoxicity increases with generation number.

Reduced cytotoxicity of amine-blocked PAMAM-g-diazirine (Figure 7) is a result of reduced exposure of fibroblast cells to a toxic effect of inherent primary amines from dendrimer structure. Moreover, the degrees of neovascularisation and fibrosis for PAMAM-g-diazirine-blk were even lower than the control, indicating lower degree of tissue reaction towards PAMAM-g-diazirine-blk than that of PLGA. However, the degree of infiltration of giant cells was reduced to none for PAMAM-g-diazirine possibly due to the germicidal ability of –NH_2_ groups [35,39]. These results indicate that the tissue adhesive warrants further investigation towards vascular patches, drug depots, and hydrogel biosensor implants [17,18,40,41,42,43,44,45,46].

## 3. Materials and Methods

Poly (amidoamine) (PAMAM, from 1st generation, G1 to 5th generation, G) were purchased from Dendritech, Midland, MI, USA and Chenyuan, Weihai, China. 3-(4-(Bromomethyl) phenyl)-3-(trifluoromethyl)-diazirine (aromatic-diazirine) was purchased from TCI, Tokyo, Japan. Methyl alcohol (MeOH), acetyl chloride and glacial acetic acid (AcOH) and THF were from TEDIA, Singapore. Poly-dl-lactide-*co*-glycolide (PLGA 53/47) from Sigma (Singapore) was used as received. Phosphate buffer saline (PBS; Gibco, Singapore) was employed in all experiments. Glass slides (25.4 × 76.2 mm, 1–1.2 mm thick) were purchased from CLP, Tianjin, China. 3T3 fibroblasts cells were from ATCC, Singapore. Dulbecco’s modified Eagle medium (DMEM) and fetal bovine serum (FBS) were both purchased from Gibco, Singapore. 24 well plates were from Thermo Fisher Scientific, Shanghai, China. Alamar Blue was from Thermo Fisher Scientific Inc., Boston, MA, USA.

G1–G5 PAMAM MeOH solution (5 mL, 5 wt %) was prepared and aromatic diazirine was added to produce conjugation percentages ranged up to 30% for G2 to G5. The nucleophilic substitution reaction took 36 h under room temperature in dark. G1–G5 PAMAM-g-diazirine was dried under vacuum, as pale yellow viscous liquid. Conjugates were stored in dark under 4 °C. Solution was kept stirred in ice bath for 1 h. Acetyl chloride was dropped into the MeOH solution in ice bath dropwise. The temperature was monitored which was kept below 10 °C. Molar ratio between –NH_2_ from PAMAM-g-diazirine and acetyl chloride was controlled as 1:5. The ‘blocked’ conjugates were precipitated out by addition of 30 mL THF after 24 h stirring in dark under room temperature.

Aromatic diazirine was prepared in MeOH with a concentration gradient (0.2, 0.4, 0.5, 0.8 and 1 mM). UV extinction coefficient of aromatic diazirine was the slope of the linear fitting curve of absorption at 350 nm against concentration. SEC-MALS-UV is connected in a line. AcOH aqueous solution (1% *w*/*v*, 166.7 mmol) with 0.2% (*w*/*v*) NaN_3_ (30.8 mM) was prepared as the mobile phase. PAMAM as the control and PAMAM-g-diazirine samples were dissolved in 1 mL of mobile phase with a concentration of 2 mg/mL. All the samples were filtered by 0.22 µm filter and sealed in 1 mL HPLC glass vials. RI, LS and UV absorption (350 nm) were obtained and processed by Wyatt ASTRA V. The *dn*/*dc* value of PAMAM was 0.185 [47]. The UV extinction coefficient of Br-diazirine in aqueous medium was measured as 1009.5 mL cm^−1^ g^−1^ at 350 nm which was used to calculate the experimental conjugation percentage. TNBS assay was performed according to the protocols provided by Thermo Fisher Scientific, but other methods may be applicable [48].

G1–G5 PAMAM, aromatic diazirine, and all PAMAM-g-diazirine were analyzed by NMR (Bruker Avance, Midland, ON, Canada) at 400 mHz with DMSO-*d*_6_ as the solvent. H1 and C13 DEPT 135 HMQC HMBC NMR spectra were collected and analyzed. The peak assignment, 2D spectrum analysis and peak integration (^1^H-NMR) were performed with SpinWorks 4.2 (Spin.works, Lisbon, Portugal).

G1–G5 PAMAM-g-diazirine and G1–G5 PAMAM-g-diazirine-blk with different generations and conjugation percentages were prepared in PBS (pH was balanced to 7.2), and there were three concentrations for each formulation (25, 50 and 75 wt %). Samples were pipetted onto the light transparent rheology testing base (P-PTD120/GL, Anton Paar, Singapore). The UV light was generated and transferred by a S1000-1B-3188 UV initiator (OmniCure, Plano, TX, USA) with 365 nm UV filter (019-01036) to the bottom of the base for activation of PAMAM-g-diazirine. The intensity of the UV light on the surface of the base was maintained at 21.3 mW/cm^2^. Dynamic mechanical analysis was assessed in real-time at 1% amplitude and 1 Hz with 8 mm diameter parallel stainless steel probe under 25 °C and the gap between probe and base was set as 100 µm. For continue activation, UV light source was turned “OFF” for 2 min in the beginning for the baseline collection. It was turned “ON” after that for 10 min. G’ and G” of the sample were recorded during the activation. For the “ON/OFF” intervals activation, UV light was turned “OFF” for 2 min in the beginning for the baseline collection. Then, it was turned “ON” for 1 min and turned “OFF” for 1 min. This cycle was repeated 5 times for each sample.

Shear adhesive performance measurements of PAMAM-g-diazirine were based on ASTM standard F2255-05 [34]. Fresh swine aorta from a local abattoir was cut into 40 × 20 mm slides. The fat on the opposite side of the aorta was removed, and the thickness of the slide was around 1 mm. The tissue with endothelial (intima) side was glued onto a glass slide using cyanoacrylate adhesive. PLGA thin films were prepared based on a previous publication [15] and cut into 40 × 20 mm squares, and these slides were glued onto glass slides with the help of cyanoacrylate. G1–G5 PAMAM-g-diazirine PBS solution (0.3 mL) with conjugation percentages and concentration were pipetted in between the PLGA film and swine aorta surface. This glass-tissue-adhesive-PLGA-glass system was sandwiched by two paper binder clips with an average force of 1.37 ± 0.25 N/cm^2^. This ‘sandwich’ structure was activated by UV light (365 nm) immediately, and the UV intensity at the surface of this structure was kept constant at 21.3 mW/cm^2^. Exact overlap area of the gel between PLGA and tissue surface was calculated by analyzing the digital photograph with the help of Adobe Photoshop CS2 (company, San Jose, CA, USA). Series Force Measurement System (Chatillon Force Measurement, Largo, FL, USA) was used for adhesive strength test with the controlled linear speed of 3 mm/min with 10 N loading cell (*n* = 5).

Cells were cultured in DMEM containing 10% PBS at 37 °C with 5% CO_2_. Cells were suspended via trypsinization and then counted and seeded on to 24 well plates. Cell number was controlled as 10,000 in each well of the plates (*n* = 4 for each sample). Cells attached to the bottom of the plates after 24 h incubation, and the medium was refreshed before the addition of PAMAM-g-diazirine. The concentrations of each formulation were as follows: 200, 1, 0.1, 0.01 and 0.001 µg/mL. G1–G5 PAMAM-g-diazirine, G1–G5 PAMAM-g-diazirine-blk conjugates were examined and the unmodified G1–G5 PAMAM were the positive controls. The viability of the cell in each well was quantitative measured via Alamar Blue assay, and the fluorescence strength of samples was measured by a microplate reader with 560EX nm/590EM nm filter settings (Infinite M200, TECAN, Männedorf, Switzerland). Values were considered to be significantly different if the *P* value was less than 0.05.

G5 PAMAM-g-diazirine (theoretical conjugation percentage is 15%) and the corresponding G5 PAMAM-g-diazirine-blk were implanted subcutaneously into Wistar female rats (300 ± 50 g, 10-weeks old, InVivos Pte Ltd., Singapore). Mice were sedated with inhalation of isoflurane (2%) and the analgesic was administered by intraperitoneal (IP) injection (tramadol, 3 mg/kg). Experiments and the protocols were approved by Nanyang Technological University Animal Care and Use Committee (IACUC; Protocol: ARF-SBS/NIE-A0301). Hair of the mice was removed. The surgical area was sterilized using povidone iodine solution, and then washed by 70% ethanol. Four wounds with 1.5 cm length and 0.5 cm depth were cut on the dorsum of each rat exposing the muscle tissue. The two conjugates mentioned in the beginning of this section was prepared in PBS solution (50 wt %) and then applied onto PLGA patches (diameter = 6 mm; thickness = 0.1 mm). These patches were implanted directly on exposed muscle tissue, and the adhesive conjugates were sandwiched in between the patch and the tissue. PLGA patches without adhesives were implanted as the negative control. The adhesives were stimulated by UV light, generated and transferred by a S1000-1B-3188 UV initiator (OmniCure) with 365 nm UV filter (019-01036) at 20% power from approximately 1 cm distance (~100 mW/cm^2^ for 1 min: 6 J/cm^2^). Then, the skin cuts were sealed by MaxonTM 3–0 sutures (the surgical procedures were demonstrated by Figure S2). Mice were kept monitored, and the body weight was recorded for one week. The two rates were sacrificed on the 7th day. The dermal suture implants, the internal implant material and the dissected samples of skin with epidermal were fixed in formalin. Entire implant site for each sample was sectioned perpendicular to the skin surface and parallel to plane of incision. Tissue was then processed by a Sakura VIP Tissue Processor (Lab X, Midland, ON, Canada) with increasing concentration of ethanol, xylene and finally paraffin. The processed tissue was embedded into paraffin block which was then sectioned into 5 µm thick slides by rotary microtome. Slides obtained were dried and stored in incubator (60 °C/15 min) before the H&E staining by an Autostainer XL (Lab X, Midland, ON, Canada). The reaction between implant material and deep dermal tissue was evaluated by a boad-certified pathologist with the help of Ariol software/Slidepath Tissue IA software (Leica Microsystems, Buffalo Grove, IL, USA). The collagen extraction was confirmed by analysis of the sections stained by Masson trichrome histochemical.

## 4. Conclusions

We have produced bioadhesive formulations based on PAMAM dendrimers conjugated with diazirine that act as the UV-activated carbene precursor for polymer crosslinking. In order to maintain adhesion strength and to reduce the cytotoxicity of our formulations, capping of the pendant amines was established. The conversion of amines to amides resulted in a lowering of the adhesion strength of our formulation, the overall properties of the blocked conjugates still have considerable advantages. In vitro compatibility of amine-capped formulation with fibroblast cells was significantly improved by reducing the concentration of cationic amine groups. Our results demonstrated higher cytotoxicity for the higher generation of dendrimers as a consequence of higher amine concentration, consistent with other investigations. G5 dendrimer displayed rapid rise in storage modulus and had the highest shear modulus observed among the formulations tested. Blocked PAMAM-g-diazirine showed improved in vitro fibroblast compatibility and reduced immunological response in vivo while maintaining excellent tissue adhesion strength (ex vivo) of 17 kPa, several times higher than that of commercially available fibrin glue.

## Supplementary Materials

The following are available online, Figure S1: 1H-NMR spectra of PAMAM and G1–G5 PAMAM-g-diazirine, Figure S2: the surgical procedures for in vivo histological analysis, Table S1: Structure characterization information for G1–G5 PAMAM-g-diazirine and G1–G5 PAMAM-g-diazirine-blk conjugates, Table S2: Rheological performance and adhesion strength of all the G5 PAMAM-g-diazirine and PAMAM-g-diazirine-blk conjugates.
