# Supplementary material for: On-Demand Bioadhesive Dendrimers with Reduced Cytotoxicity
Source: Molecules. 2018 Mar 30;23(4):796. doi: 10.3390/molecules23040796 (PMC6017702; doi:10.3390/molecules23040796)
Supplement: Supplementary file 1 [file molecules-23-00796-s001.pdf]

molecules

Supplementary information

On-demand bioadhesive dendrimers with reduced cytotoxicity

Feng Gao [1], Ivan Djordjevic [2], Oleksandr Pokholenko [3], Haobo Zhang [1], Junying Zhang [1,*] and Terry W.J. Steele [3,*]

[1] School of Material Science and Engineering, Beijing University of Chemistry Technology, North Third Ring Road 15, Chaoyang District, Beijing 100029, China; gaofeng@mail.buct.edu.cn (F.G.); zhanghaobo9093@163.com (H.Z.)

[2] Escuela de Ingeniería y Ciencias, Tecnologico de Monterrey, Ave. Eugenio Garza Sada 2501, Monterrey 64849, NL, Mexico; idjordjevic@ntu.edu.sg

[3] School of Materials Science and Engineering, Division of Materials Technology, Nanyang Technological University, Singapore 639798, Singapore; opokholenko@ntu.edu.sg

* Correspondence: **wjsteele@ntu.edu.sg (T.W.J.S.); zhangjy@mail.buct.edu.cn (J.Z.)** Tel: +65-6592-7594 (T.W.J.S.); +86-10-6442-5439 (J.Z.)

Table S1: Structure characterization information for G1-G5 PAMAM-g-diazirine and G1-G5 PAMAM-g-diazirine-blk conjugates.

			**PAMAM-g-diazirine**					**PAMAM-g-diazirine-blk**	
**generation**	**initial molar mass (Da) [a]**	**Theoretical diazirine conjugation percentage**	**eluent volume (mL)**	**molar mass calculated by eluent time (Da) [b]**	**molar mass calculated by multiple angles light scattering (MALS) (Da) [c]**	**Diazirine conjugation percentage calculated by NMR [d]**	**Diazirine conjugation percentage calculated by RI and UV signal [e]**	**Residual –NH_2 percentage calculated by molecular weight increase (MALS) [f, g]**	**Residual –NH_2 percentage measured b TNBSA[f]**
G1	1430	0	7.621	1430	2700 ± 300	0 ± 0.54	0.0 ± 0.3%		
		37.5	7.138	4073	2200 ± 154	38.4 ± 0.54	33.4 ± 2.3%	25 ±8.8%	8.8 ±0.33%
		62.5	6.988	5303	2100 ± 170	68.2 ± 0.54	51.9 ± 4.5%	4 ±5.2%	5.2 ±0.54%
G2	3250	0	7.071	3250	4300 ± 260	0 ± 0.54	0.0 ± 0.2%		
		20	6.905	6165	3900 ± 274	22.7 ± 0.54	15.6 ± 1.1%	20 ±8.4%	8.4 ±0.69%
		30	6.796	7508	4200 ± 337	29.6 ± 0.54	22.4 ± 1.8%	10 ±7.4%	7.4 ±0.82%
G3	6909	0	6.863	6909	7300 ± 293	0 ± 0.54	0.0 ± 0.1%		
		10	6.771	7874	8200 ± 409	11.4 ± 0.54	14.2 ± 0.7%	22 ±5.3%	5.3 ±0.44%
		20	6.705	8890	8500 ± 255	21.8 ± 0.54	17.6 ± 0.5%	15 ±7.3%	7.3 ±0.16%
		30	6.696	9043	8100 ± 324	33.2 ± 0.54	28.7 ± 1.2%	16 ±3.9%	3.9 ±0.64%
G4	14215	0	6.496	14215	14600 ± 439	0 ± 0.54	0.0 ± 0.2%		
		10	6.413	15467	15500 ± 311	12.3 ± 0.54	10.2 ± 0.2%	10 ±14.2%	14.2 ±0.36%
		20	6.404	15745	15400 ± 615	21.1 ± 0.54	21.1 ± 0.8%	20 ±12.3%	12.3 ±0.29%
		30	6.389	16220	16800 ± 168	32.4 ± 0.54	28.4 ± 0.3%	20 ±13.1%	13.1 ±0.69%
G5	28826	0	6.096	28826	27600 ± 552	0 ± 0.54	0.6 ± 0.3%		
		10	6.012	32011	33200 ± 663	14.6 ± 0.54	13.2 ± 0.6%	14 ±6.8%	6.8 ±0.35%

20	5.917	39063	32000 ± 1293	22.5 ± 0.54	19.8 ± 0.8%	19 ±9.1%	9.1 ±0.44%
30	5.884	41892	35000 ± 1054	31.7 ± 0.54	29.6 ± 1.1%	2 ±2.7%	2.7 ±0.61%

[a] This is the theoretical molar mass of unmodified PAMAM for each generation

[b] Commercially available PAMAM from G1 to G5 were used as the SEC M_w standards

[c] For this light scattering analysis, dn/dc value used here was 0.185

[d] The total injection mass of dendrimer was calculated via integration of refractive index signal area. The mass of diazirine was calculated from the UV absorbance signal and the UV extinction coefficient of diazirine. (1008 mL g^{-1} cm^{-1})

[e] peak a and f (**Figure 4**) were chosen as the standards to calculate the amount PAMAM and diazirine respectively.

[f] Percentage values are the percentage of residual $–NH_2$ after the ‘blocking’ reaction by acetyl chloride.

[g] Values calculated from the increase of molecular weight measured via SEC-MALS-UV system as well as the tested diazirine conjugation percentage values.

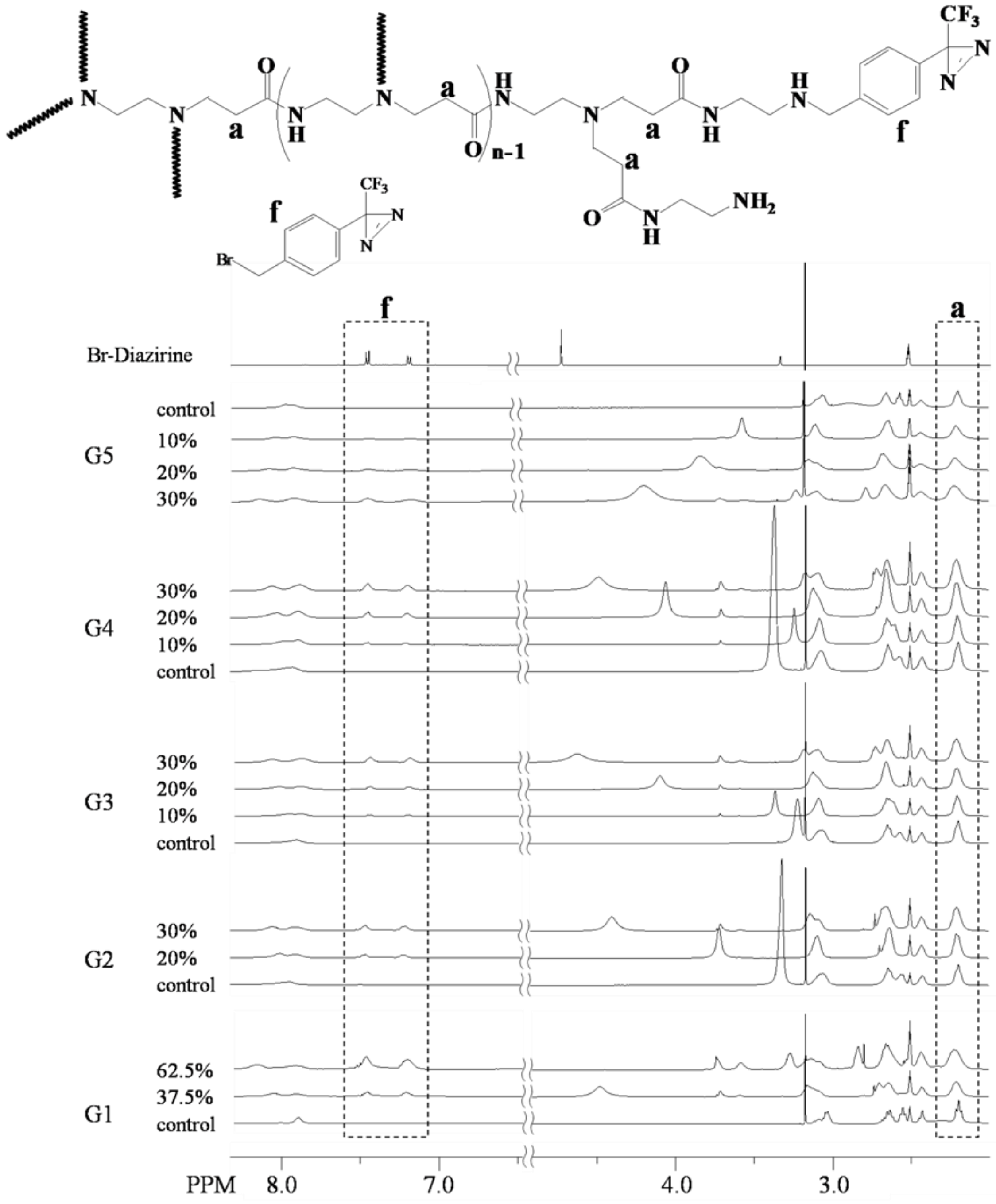

Figure S1. ^{1}H NMR spectra of PAMAM and G1-G5 PAMAM-g-diazirine.

Table S2: Rheometry results and adhesion strength of G5 PAMAM-g-diazirine and PAMAM-g-diazirine-blk conjugates.

			PAMAM-g-diazirine			PAMAM-g-diazirine-blk		
generation[a]	Theoretical diazirine conjugation degree (%)	Concentration (wt%)[b]	G' after 2 min stimulation (kPa)	G' after 5 min stimulation (kPa)	ex vivo adhesion strength towards fresh aorta (kPa)	G' after 2 min stimulation (kPa)	G' after 5 min stimulation (kPa)	ex vivo adhesion strength towards fresh aorta (kPa)
3	10	25	-[c]	-	-	-	-	-
		50	0.061	0.063	0.2	0.047	0.049	0.17
		75	0.13	0.14	0.48	0.1	0.11	0.36
	20	25	-	-	-	-	-	-
		50	0.083	0.097	0.3	0.077	0.074	0.27
		75	0.57	0.61	0.3	0.43	0.45	0.3
	30	25	-	-	-	-	-	-
		50	0.17	0.16	0.21	0.15	0.16	0.33
		75	7.3	9.2	3.2	6.5	7.4	0.17
4	10	25	0.43	0.41	0.36	-	-	-
		50	0.93	1.08	1.6	0.82	0.87	1.1
		75	1.22	1.45	1.4	0.9	1.43	1.5
	20	25	0.56	0.78	0.3	0.44	0.48	0.3
		50	3.6	4.2	1.87	3.3	3.4	1.3
		75	1.32	1.36	1.74	1.21	1.2	1.9
	30	25	1.3	1.1	1.2	1.2	1.6	1.5
		50	6.2	6.4	3.3	5.7	6.3	2.1
		75	7.8	11.2	7.4	4.6	9.7	3.2

		25	2.5	2.4	1.8	2.4	2.2	1.4
	10	50	21	27	5.3	18	21	3.5
		75	27	54	4.3	26	38	3.6
		25	22	31	2.3	21	29	2.2
5	20	50	77	94	11.2	69	81	9.7
		75	86	108	14.3	65	79	11.2
		25	44	41	8.2	36	39	4.5
	30	50	87	143	21	63	114	15.4
		75	164	225	25.3	95	151	17.1

[a]: Conjugates based on G1 and G2 were not listed since there was no crosslinking observed for these formulations.

[b]: all the adhesives were prepared in PBS solution.

[c]: '-' listed in the table above indicated there was no crosslinking observed for the corresponding formulation.

molecules

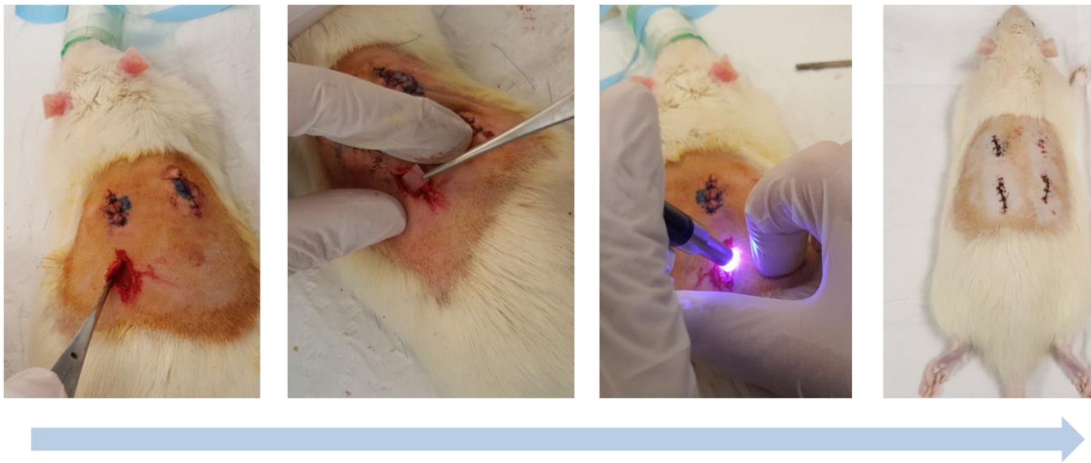

Figure S2: Surgical procedure for subcutaneous implantation and subsequent UV-activated crosslinking of bioadhesive formulations.
